# Medical Notes and Observations

**Published:** 1849-05

**Authors:** Caleb Green

**Affiliations:** Homer, N. Y.


ART. II.—*Medical Notes and Observations.* By CALEB GREEN, M. D., of Homer, N. Y.

If the following, which are a few of the items of medical experience and observation which have been noted in my case-book within a few months back, will be of any interest or value to the medical public, I shall consider myself amply rewarded for having recorded them.

*Variola Vaccina, commencing* 12 *days after Inoculation.*—June 1st, 1848, I vaccinated Francis S., æt. about 47 years. After waiting the ordinary time, the virus did not produce its usual effects, and I suspected, of course, that it was not good, or had not been properly introduced. On the the 12th day, however, the papular stage commenced, and proceeded to the well formed vaccine pustule, attended with strong febrile symptoms, and considerable swelling of the arm.

---

*Case of Mrs. O.*—July 17, 1848. Was called this evening to see Mrs. O; a lady æt. 35 or 40 years, of the nervo-bilious temperament, who had just experienced a sudden swelling of the upper lip, particularly of the left side, with active congestion of the mucous membrane of the left compartment of the nose. Lancinating pains darted from the lip toward the left eye, and there was a prickling sensation of the tongue—whether of the whole of that organ, or only one side, I neglected to note. She assured me that these symptoms all appeared within three or four minutes from the commencement of the attack. She had been at work in an outbuilding, but not exposed to a current of air. The tumefaction was not painful, but felt inconveniently. I did nothing for it. July 18th, the swelling and other symptoms have disappeared. In what system of nosology has this combination of symptoms " a local habitation and a name ? " Is this one of the protean shapes of hysteria?

---

*Coffee.*—Mrs. A. informs me that she drinks coffee for the purpose of preventing constipation. She suffers much from menorrhagia and headach. Pereira (Mat. Med. and Therap., vol. 2, p. 462) refers to two cases in which it operated as a purgative. He says, however, that it is apt to have a constipating effect on those unaccustomed to its use. Dr. Dick also (Bost. Med. & Surg. Jour. vol. 36, p. 332) says it sometimes constipates. Is coffee a *stimulant* or a *sedative?* Pereira (op. cit. vol. 1, p. 94) says that it is a tonic and stimulating drink. Dick (Bost. Jour. loc. cit.) says that it exhilarates and invigorates without hurtfully stimulating; that it has a peculiar effect on the cerebral organs, promoting in a more remarkable degree than, perhaps, any other drink, the normal action of the intellectual and imaginative faculties. I have watched its operation on myself, and find that a tolerably strong decoction immediately excites the action of the heart.

On the other hand, Billing (First Prin. Med. p. 101) contends for its sedative qualities. His error is committed by considering the action of *tea* and coffee as identical, whereas, although they both produce wakefulness and mental exhilaration, the one is a cardiac sedative, and the other a cardiac stimulant. I can, by no means, agree with Billings' view of the modus operandi of tea and coffee in producing cerebral excitement, even should I admit both of them to be sedatives. His opinion is, that they operate by counteracting the plethoric state of the brain by virtue of cardiac sedation. If this were true, tartarized antimony should do the same, as well as many other medicinal sedatives, and blood-letting. The truth is, both tea and coffee are cerebro-spinants or " medicines which produce or prevent sleep, or which affect the intellectual functions, &c.," (Pereira *op. cit.*) while, at the same time, their action on the heart and arteries may be diverse. The peculiar principles of both tea and coffee are developed during torrefaction. —(See *Brit. and For. Med. Chi. Rev.*, July, 1848, and *Pereira op cit.*)

---

*Great Nervous Impressibillity from severe Hæmorrhage.*—July 3d, 1848. Mr. Herbert informs me that he can predict a storm two or three days before its arrival, by his sensations, and that this faculty has existed only a year. This condition of the system he attributes to the loss of large quantities of blood, in consequence of severe injuries he received a year since. Never felt the effects of an approaching storm before that severe hæmorrhage. Cannot bear warm weather now as well as before.

---

*Tradescantia* versus *the Venom of the Rattle snake.*—July 12th. A friend communicates to me the fact that the wiseacres of Wisconsin pretend to cure the bite of the rattle snake by the root of the tradescantia or spider lily.

*Metastasis of active Congestion of the Bronchia to the Fauces and Nasal Cavities.*— July 6th, 1848. Mr. M. related to me his case, which he termed "a cold." Said he yesterday suffered from great "soreness of the lungs." Soreness particularly extending down the sternum, and somewhat throughout the chest generally. The respiration was painful; cough dry; thought he had absolute inflammation of the lungs. Was in much distress from this condition last night when he retired, but fell asleep, from which, in the course of the night, he awoke suddenly, experiencing a most delightful sensation of relief from any abnormal pulmonary symptoms, but found the fauces and nasal cavities, which, on retiring, were perfectly free, in a state of active congestion—that he had "a cold in the head." This state of things still continues; lungs free. July 8th, says he is entirely free from his cold; has pursued no treatment. A slight epidemic of influenza is now prevalent, and this is probably a case of it. He was wholly unable to account for the cold. I ought to have mentioned that he suffered with *hemicrania.*

*Tincture of Nux Vomica in Cephalalgia.*—June 7th, 1848. In conversation with my friend Dr. Hawley, of Ithaca, on the subject of hobbies and specifics in medicine, he observed that he rode one hobby, and that was the saturated tincture of nux vomica. He uses it freely in those cases popularly termed "sick headach," and also in those forms of cephalalgia which appear to be of a decidedly neuralgic character. He remarked that he had very gratifying success in the treatment of these affections with this agent. During the paroxysms he usually administers from 15 to 20 drops, which generally gives relief; after which he often continues the tincture in doses of 10 or 15 drops three times a day for two or three days. He assures me that the first sometimes produces permanent relief; and the repeated doses frequently protect the patient almost wholly from future attacks.

Since the above date I have employed the tincture in several cases of cephalalgia, and with various success. The following is my formula for the tincture. ℞. Nux vom. pulv. ℨi; spts. vin. rect. ℥iv. M. ft. tinct.

June 15. Mrs. Blanque has been afflicted for several weeks with a severe headach, which is evidently sympathetic of gastric and uterine derangement. Gave her, this P. M., 15 drops of tr. nux vom. She said that within ten minutes after taking the medicine, she had a "queer sensation all over her," that her head felt airy and light, and "as though she wanted to fly away;" but did not describe the symptoms as decidedly unpleasant. The headache was relieved. June 16, this morning she remarked to me " how good it seems to be free from headache! My head is clear, and does not ache at all." For five or six weeks previously she had an almost *constant* headache during the day. June, 23d, says her head feels very well, and has had so much relief from the remedy that she wants a vial of it for future use, if necessary. June 29, says she now has severe pain in the back when she does not have it in the head. Took the tincture with relief.

June 28. Mrs. P. has been complaining for some time of a peculiar dull congestive headache, attended with heat and redness of the scalp, just anterior to the "organ of firmness," covering a space an inch and a half, or two inches in diameter. The pain extends over most of the head, but is more severe in the upper and anterior portions. There is sometimes amaurosis and nausea. She is of a thin habit, pale and habitually constipated. This morning was suffering much with headache, amaurosis, loss of appetite, &c. Gave her 20 drops of the tincture, and in fifteen or twenty minutes her head was greatly relieved and appetite improved. Had continued pretty well during the day.

July 3d. Mrs. N., æt. about 35, of the nervo-bilious temperament, sent for me about 8 P. M. in great haste. Found her suffering from severe cephalalgia, lancinating pain over the right eye, with great restlessness, nausea and vomiting. Gave 20 drops of the tincture. In twenty minutes became easier, nausea and vomiting ceased, and she rested very well during the night, which was quite contrary to her expectations. July 4th, complains of some heaviness of the head, but is otherwise free from pain.

July 18. L. S., æt. about 13 years, had suffered for two or three months with a dull headache every day—continuing nearly through the day. I have tried medication with blue mass, laxatives, vegetable tonics, &c., without any marked amendment. Three or four weeks since, I commenced giving him the tincture of nux vomica, soon after which he began to amend. He took ten drops twice a day for 10 or 15 days. He is now entirely free from headache. He is of the nervo-bilious temperament, and his father, and some other members of the family, suffer from the sick headache.

March 7th, 1849. Rev. Mr. F., of the nervo-sanguine temperament, suffers frequently from headache, with nausea. The cephalalgia is sympathetic of gastric disturbance, and is principally characterized by sharp pain over the right eyebrow. Sometimes extending to the temporal region. He has taken the tincture with some relief, although it sometimes leaves that enlarged, light sensation in the head, alluded to in the case of Mrs. Blanque. He takes it in doses of 10 or 12 drops, repeated not oftener than once a week, and it operates as a laxative. This, it will be remembered, is a favorite remedy of the homœopathists for constipation, but, in order to have its due effect, it must be given in something more than the "diluted starlight" doses.

In the Amer. Jour. Med. Science, Jan., 1849, p. 34, Dr. Porter relates a case of headache attended with indistinctness of vision, numbness of the right side of the tongue and right arm, occurring in a patient on the third day after confinement, in which there were signs of debility rather than of congestion, and in which he administered tinct. nux vom. with evident happy effect, not only on the local symptoms, but as a tonic to the stomach. He also alludes to some other cases in which these amaurotic symptoms, succeeded by headache, and this by numbness of the tongue and arms and nausea, suddenly supervening during gestation or soon after labor, and always in connection with gastric disturbance, were promptly relieved by tincture nux vomica.

---

*Fracture of the Clavicle.*—Mr. Herbert, a teamster, on the 29th of May, when near Syracuse, thirty miles distant, fell from his wagon, struck upon his left shoulder, producing a fracture of the clavicle, of which latter fact however, he was not at the time aware, although the arm was useless, and he was unable to raise the hand to the head. He put his arm in a sling, went on to Syracuse, obtained a load of goods, and returned the next day. On the 31st he came to me for examination, when I found a fracture at the usual point, the external extremity of the internal curvature of the clavicle. I immediately reduced the fracture, and dressed it with a combination of Boyer's and Fox's apparatus. Notwithstanding the most careful and firm adjustment of the apparatus, a displacement of the fragments to the extent of two or three lines, was not avoided. The patient wore the confining apparatus about three weeks, when he removed it, leaving only the sling. The callus had much diminished, but I still advised him to keep the parts confined. He did not, however, and at the end of five weeks took off his sling, put on his coat and went to work, contrary to my advice. The fracture remained united and sound.

---

*Collodion.*—I need not detail the process of preparing this new adhesive agent, as it is given in full in the number of this Journal for August last. I will merely state that I find by experiment no advantage in macerating the cotton in the acids more than *thirty minutes.* I have allowed it to remain *twelve hours,* but it was not superior to that exposed to the acids only half an hour. I find also that the ether should be perfectly fresh in order to make a solution of the prepared cotton. The bottle containing it should not be frequently opened, and should be kept very tightly stopped. Without these precautions, practitioners will fail in the preparation of collodion.

I have used this agent repeatedly in *burns* and *scalds* with the most prompt and happy effect. Where, however, the cuticle is abraded and serum is exuding, the collodion will not adhere. In *wounds,* after the arrest of hemorrhage, it has been used with excellent success. Water dressings can be used more freely than when other modes of adhesion are practiced, but in the end the collodion straps will loosen. In *herpes labialis, acne,* and some other *cutaneous eruptions,* I have employed it with marked advantage. In *chilblains* I have used it with the most decided success. In one case the patient had her feet for some time exposed to severe cold, which produced an erythematous inflammation of several of the small toes. They were swollen, red, tender, and itching. I completely enveloped them in a thick coating of collodion, and the contraction which took place on its drying produced so much compression of the vessels, that the toes were as pallid as frozen ones. The pain and itching were immediately relieved, and in twenty-four hours these members were nearly well. I have cured pernio of the heel, also, with this article, but I do not believe it a panacea for all cases of chilblain, even in its erythematous stage.

*Observations on the Etiology of Dysentery.*—Our village in August and September last, suffered from the prevalence of dysentery, but it was in the main confined to a small portion of the village—the northern third of Main Street. This street runs nearly parallel with the river, and on an average, about thirty rods from it. The river in the upper third of the village is converted into a mill-dam, which skirts the village lots on the east. On the eastern side of the pond, a hill rises pretty abruptly to the heighth of three or four hundred feet. The pond, late in the summer, becomes in a measure stagnant, from deficiency in the supply of water. It was just in this portion of the village that the dysenteria first made its appearance and prevailed most extensively. Indeed the prevalence and virulence of the disease was in a very great degree limited by the extent of the pond. Hardly a house escaped on both sides of the street opposite the mill-dam. In some houses several were sick at a time, and most of the fatal cases—which, fortunately, were but few—occurred in that district;—in fact but one death, that I now recollect, occurred out of it, and that was on Factory Street, which joins the upper part of Main at an acute angle opposite the lower end of the pond. The families in this part of the village, with perhaps a single exception, are well fed, clothed and regulated;—in short, they are the "up town" class, and none of the causes of malignant fever or dysentery existed in their domestic arrangements or habits. A few other cases were scattered over the other portions of the village and town, but were mild when compared with those alluded to. The intelligent reader can draw the proper inferences in reference to this matter, in which he may be assisted by consulting Dr. Gayely's article on the "Etiology of Intermittent and Remittent Fevers," in the January No. of the Am. Jour. Med. Science, p. 66–67.

---

*Post Mortem Appearances of Dysentery.*—The subject of this note—Wm. R. R. æt. 18 months—was attacked on the evening of the 29th of August, 1848, with diarrhœa of a bilious character. I did not see him until the next day at 5 o'clock, P. M., when I found him very restless, with frequent but not copious stools, consisting of a greenish mucus, with a little blood. There was considerable tenesmus at each evacuation. After the first day of treatment the discharges did not present any blood. On the 4th and 5th days of the disease the evacuation had a feculent portion of a dark brown color, somewhat resembling coffee grounds. Cerebral disturbance began to manifest itself on the third day, and for the last three days the patient was for the most part comatose. The brain was very largely developed when compared with the body. He was cutting some of his teeth. On the fifth day he passed the chalky urine spoken of by Coindet. He died on the morning of Sept. 7th.

*Autopsy*, 24 hours post mortem.—Dr. Bradford assisted in the inspection. The abdominal and pelvic viscera only were noticed. The position of the organs was normal. The stomach, liver, and small intestines, were healthy in appearance, with the exception of the last five or six inches of the *ileum*. From this point to the lower end of the rectum the results of inflammation were very manifest. The muscular coat was much thickened, and the mucous coat nearly covered with an exudation of lymph which was yellowish in the upper portion of the colon, but had a white and "curdy" appearance in the rectum. Between the patches of lymph the mucous membrane was of a dark color. There was no ulceration. The meso-colon and meso-rectum were morbibly vascular. The kidneys and bladder were healthy in appearance.

---

*Singular Effects of Hydriodate of Potash.*—In July, 1848, I gave Mr. William Dalrymple, farmer, a solution of hyd. pot. of about twenty grs., to the ounce of water, and directed the use of a teaspoonful twice a day. He took the first dose, and in about five minutes afterwards began to experience feelings of intoxication. He remarked that his head felt large, and he "felt large all over;" had vertigo and experienced the usual reckless feelings of inebriation. He is satisfied that this was the result of the hyd. pot. Since, the same symptoms were excited on a repetition of the dose. I have never before observed the effects of hyd. pot. above referred to, nor met with the remarks of those who have witnessed them. headache, watchfulness, and other symptoms indicative of the action of the salt on the nervous system, have been noticed by Drs. Clendinning and Wallace.

---

*Nitrate of Potash alias Nitrate of Silver.*—Two or three months since I purchased a stick of "pure" Nit. Silver of our village druggist, who usually keeps medicines of excellent qualities. I observed that it had not the external appearance which that salt usually has, it being *very* white and easily broken down into small irregular crystals. I used it several times for conjunctivitis, but without the usual effects of Nit. Argt.. or rather no effect at all. I was led to examine the article more carefully, and found by taste, burning, and chemical tests, that it was a *very* pure specimen of *nitrate of potash*—not a trace of silver to be detected. The other sticks in the bottle from which this was taken, consisted of *pure* nitrate of silver —as good as the crystallized salt.

---

*Remarkable Case of Abstinence.*—In Dec. 1848, I visited, in consultation with Drs. Goodyear, Hyde and Bradford, a Mr. Gifford, of Groton, Tompkins Co., who had been insane for several months, but for the three weeks previous to our visit—laboring under the impression that his friends wished to poison him—had eschewed all kinds of solid nourishment, and had taken nothing into his stomach during that time but cold water and snow. He usually drank the water twice a day, drawing it himself directly from the well, but after the fall of snow he resorted to that. He had not manifested any inconvenience or debility from this abstinence until the afternoon of our visit, when it was evident that he had been using a larger quantity of snow than usual, and we found that he had vomited a moderate quantity of watery fluid tinged with bile and a little blood, and was manifesting some internal pain, from which, however, he recovered before we left. He was a large muscular man, and his strength and flesh did not appear to be at all diminished. By our advice a stratagem was resorted to, in order to induce him to leave his quarters and take food. It was successful. He went off with a neighbor, and after an abstinence of *thirty three days* he took some *toast water*, and on the thirty-fourth, some toast, and after that took full and regular meals, without any apparent inconvenience. He relapsed, once or twice, into his fear of poison, but was soon induced to resume his meals. There was no apparent loss of strength, or flesh, at the end of this long fast. He is now in the Asylum at Utica. Some years ago I knew an insane patient in Onondaga Co., who abstained from food entirely for twenty-one days, and terminated the fast by eating a tin pan full of snow.

But my budget is full. I may send you another of "the same sort" "in the course of time."

HOMER, March, 16th 1849. C. GREEN.

